# Safety and efficacy of nintedanib as second-line therapy for patients with differentiated or medullary thyroid cancer progressing after first-line therapy. A randomized phase II study of the EORTC Endocrine Task Force (protocol 1209-EnTF)

**DOI:** 10.3389/fendo.2024.1403687

**Published:** 2024-06-27

**Authors:** Sophie Leboulleux, Ellen Kapiteijn, Saskia Litière, Patrick Schöffski, Yann Godbert, Patrice Rodien, Barbara Jarzab, Domenico Salvatore, Sylvie Zanetta, Jaume Capdevila, Lars Bastholt, Christelle De La Fouchardiere, Yassine Lalami, Stéphane Bardet, Frank Cornélis, Marek Dedecjus, Thera Links, Ward Sents, Martin Schlumberger, D. Laura Locati, Katie Newbold

**Affiliations:** ^1^ Department of Nuclear Medicine and Endocrine Oncology, Gustave Roussy and Université Paris Saclay, Villejuif, France; ^2^ Department of Medical Oncology, Leiden University Medical Center, Leiden, Netherlands; ^3^ European Organisation for Research and Treatment of Cancer (EORTC) Headquarters, Brussels, Belgium; ^4^ Department of General Medical Oncology, University Hospitals Leuven, Leuven Cancer Institute, KU-Leuven (KU), Leuven, Leuven, Belgium; ^5^ Department of Oncology and Department of Nuclear Medicine, Institut Bergonie, Bordeaux, France; ^6^ Department of Endocrinology, Diabetology and Nutrition, Angers University Hospital Center (CHU) d’Angers, Angers, France; ^7^ Department of Nuclear Medicine and Endocrine Oncology, Maria Sklodowska-Curie National Research Institute of Oncology Gliwice Branch, Gliwice, Poland; ^8^ Department of Public Health, Azienda Ospedaliera Universitaria “Federico II”, Napoli, Italy; ^9^ Department of Medical Oncology, Centre Georges-Francois-Leclerc, Dijon, France; ^10^ Vall Hebron University Hospital, Vall Hebron Institute of Oncology (VHIO), Barcelona, Spain; ^11^ Department of Oncology, Odense University Hospital, Odense, Denmark; ^12^ Department of Medical Oncology, Centre Leon Berard, Lyon, France; ^13^ Institut Jules Bordet/Hôpital Universitaire de Bruxelles (HUB), Anderlecht, Belgium; ^14^ Department of Nuclear Medicine and Thyroid Unit, Centre Francois Baclesse, Caen, France; ^15^ Department of Medical Oncology, Cliniques Universitaires Saint-Luc, Brussels, Belgium; ^16^ Maria Skłodowska Curie’s National Institute of Oncology, National Research Institute, Warsaw, Poland; ^17^ University Medical Center Groningen, University of Groningen, Groningen, Netherlands; ^18^ Gustave Roussy, Department of Nuclear Medicine and Endocrine Oncology, Gustave Roussy and Université Paris Saclay, Villejuif, France; ^19^ Head and Neck Medical Oncology, Fondazione IRCCS, Istituto Nazionale dei Tumori, Milan, Italy; ^20^ Head and Neck Unit, Royal Marsden Hospital Sutton Surrey, Surrey, United Kingdom

**Keywords:** nintedanib, RAIR DTC, MTC, phase II trial, triple-angiokinase inhibitor

## Abstract

**Background:**

Nintedanib is a triple-angiokinase inhibitor with potential activity in patients with advanced thyroid cancers, as radioiodine refractory differentiated thyroid cancer (RAIR DTC) and medullary thyroid cancer (MTC).

**Design:**

EORTC-1209 (NCT01788982) was a double-blind randomized (2:1 ratio) placebo-controlled phase II, multi-cohort study exploring the efficacy and safety of nintedanib in patients with progressive, locally advanced, and/or metastatic RAIR DTC and MTC. The primary endpoint was progression-free survival (PFS) in the per-protocol (PP) population for both cohorts. Secondary endpoints included response rate, duration of response, overall survival (OS), and safety.

**Results:**

RAIR DTC cohort: Seventy out of the 75 planned patients with RAIR DTC (median age, 66 years; 39 women) who had progressed after one (76%) or two lines (24%) of previous systemic therapy were randomized to receive either nintedanib (N = 45) or placebo (N = 25). Of these, 69 patients started treatment and 56 met all inclusion criteria (PP). At data cutoff, the median duration of follow-up was 26.3 months in the nintedanib arm and 19.8 months in the placebo arm. In the PP population, the median PFS was 3.7 months [80% confidence interval (CI), 1.9–6.5] in the nintedanib arm and 2.9 months (80% CI, 2.0–5.6) in the placebo arm (HR = 0.65; 80% CI, 0.42–0.99; one-sided log-rank test P = 0.0947). No objective response was observed. The median OS was 29.6 months [80% CI, 15.2–not reached (NR)] in the nintedanib arm and not reached in the placebo arm. Grade 3–4 adverse events of any attribution occurred in 50% of patients receiving nintedanib and in 36% of patients receiving placebo. MTC cohort: Thirty-one out of the 67 planned patients with MTC (median age, 57 years; eight women) who had progressed after one (68%) or two (32%) lines of previous systemic therapy were randomized to receive either nintedanib (N = 22) or placebo (N = 9). Of these, 20 patients (15 in the nintedanib arm and five in the placebo arm) started treatment and met all inclusion criteria (PP). The median PFS was 7.0 months (80% CI, 1.9–8.7) in the nintedanib arm and 3.9 months (80% CI, 3.0–5.5) in the placebo arm (HR = 0.49; 95% CI, 0.16–1.53). No objective response was reported. The median OS was 16.4 months (80% CI, 12.1–24.9) in the nintedanib arm and 12.3 months (80% CI, 7.1–NR) in the placebo arm. Grade 3–4 adverse events of any attribution during the blinded period occurred in 59.1% of patients receiving nintedanib and in 33.3% of patients receiving placebo.

**Conclusion:**

This study did not suggest a clinically significant improvement of PFS with nintedanib over placebo in patients with pretreated RAIR DTC and MTC.

## Background

In 2014, at the initiation of the EORTC 1209 study, multikinase inhibitors (MKIs) were the established systemic treatment for patients with radioactive iodine refractory (RAIR) differentiated thyroid cancer (DTC) and with medullary thyroid cancer (MTC). Antiangiogenic agents (e.g., Llenvatinib and sorafenib) were the first compounds approved by regulatory authorities for RAIR DTC, with lenvatinib recommended as the primary treatment option (1, 2), whereas no second-line therapies were available yet. Antiangiogenics demonstrated a significant improvement in progression-free survival (PFS) compared to placebo: lenvatinib showed 18.3 months versus 3.6 months, and sorafenib showed 10.8 months versus 5.8 months, with both drugs still being recommended for RAIR DTC (2).

In most patients with advanced thyroid cancer, disease progression is common. After receiving initial or subsequent systemic treatments, numerous individuals can experience prolonged survival periods. This highlights the crucial need to explore alternative and efficacious therapies for such patients following disease progression. At the start of the trial in 2014, there were no approved second-line or third-line treatments for advanced thyroid cancers. Among MKIs under development in 2014, nintedanib, a MKI targeting Vascular Endothelial Growth Factor Receptor 1-3 (VEGFR 1–3), Fibroblast Growth Factor Receptor 1-3 (FGFR 1–3), and Platelet-Derived Growth Factor Receptor alpha and beta (PDGFR α and β), shows promise due to its receptor targeting, which are all overexpressed in thyroid cancer. Preclinical studies indicate its potential in inhibiting angiogenesis and tumor growth. This distinguishes nintedanib as a strong candidate for second-line treatment in RAIR DTC and MTC. A phase 2 trial (NCT01788982) was conducted to evaluate nintedanib’s efficacy in patients with advanced thyroid cancers previously systemically treated with MKIs.

## Materials and methods

### Trial design

The EORTC-1209 study was a double-blind, randomized (2:1 ratio), placebo-controlled phase II study exploring the efficacy and safety of nintedanib as second- or third-line therapy for patients with locally advanced or metastatic RAIR DTC and MTC. Patients were assigned centrally using a block design technique stratifying by country and by disease stage (locally advanced vs. metastatic). Each patient’s eligibility was assessed locally by investigators. Any deviations from the inclusion criteria outlined in the protocol were later reviewed retrospectively by the study coordinator using medical data reports, and patients were then determined to be eligible or ineligible based on these deviations.

### Trial oversight

Prior to trial screening procedures and inclusion to the study, participants were required to provide a written informed consent. The study adhered to the principles outlined in the Declaration of Helsinki and followed Good Clinical Practice Guidelines. Sponsored by the European Organization for Research and Treatment of Cancer (EORTC), the protocol received approval from the institutional review board or research ethics committee at all participating centers before the trial was activated.

### Patients

Patients meeting the following criteria were eligible for enrollment in the study: at least 18 years old, histologically confirmed DTC or MTC, locally advanced or metastatic disease considered incurable by surgery, radiotherapy and/or RAI, and one or two prior lines of systemic treatment. Additionally, they needed to have documented progression within 12 months from randomization as assessed by the local investigator using RECIST1.1. Patients had to present no evidence of active bleeding or bleeding diathesis, and a life expectancy > 12 weeks, a performance status of 0–1, the absence of uncontrolled hypertension, no history of cardiac disease within the past 12 months, no prolongation of corrected QT interval (QTc) > 480 ms, no history of other malignancy < 5 years (except for *in situ* carcinoma), and adequate general functions. Main exclusion criteria included therapeutic anticoagulation and anti-platelet therapy, history of significant gastrointestinal disorders, surgery known to affect the absorption of the drug, known intraluminal metastatic lesions with risk of bleeding, and current severe uncontrolled disease.

### Treatment

Patients started treatment within 2 days of randomization in a blinded manner. Nintedanib was orally administered at a dose of 200 mg twice daily, whereas the comparator treatment consisted of placebo capsules. Treatment was administered until documented disease progression, unacceptable toxicity, or patient refusal, with treatment cycles defined as 4-week periods. Following investigator-documented disease progression, patients and physicians were unblinded, and patients who had initially received placebo were offered the option to receive nintedanib.

Dose modifications in case of adverse events (AEs) included a decrease to 150 mg and 100 mg twice daily, temporarily stopping treatment for up to 3 weeks or permanent discontinuing treatment. There was no option for dose reescalation.

### Objectives and assessments

The primary endpoint of this study was PFS according to RECIST 1.1 investigator assessment in the per-protocol (PP) population of each cohort. Secondary endpoints were response rate according to RECIST 1.1, duration of response, overall survival (OS) in the PP population, and safety profile in the safety population (i.e., patients starting at least one dose of their allocated treatment). Sensitivity analysis included PFS, response rate, and OS in the intention-to-treat (ITT) population.

Tumor assessments were performed using spiral CT scan at baseline and every 8 weeks until progressive disease (PD). PFS was defined as the interval between the date of randomization and the date of disease progression or death, whichever occurred first. OS was defined as the interval between the date of randomization and the date of death from any cause. In the absence of an event, the patient was censored at the last date known to be alive.

AEs were reported following the National Cancer Institute Common Terminology Criteria (NCI-CTCAE) version 4.0. Safety assessments were performed throughout the study. The relative dose intensity of nintedanib was calculated as the ratio of the observed dose intensity (defined as total dose administered divided by number of days on treatment) to the dose intensity indicated in the protocol (200 mg/b.i.d.).

### Statistical methods

A 2:1 randomization was used with double of patients in the experimental arm. It was assumed that median PFS in the control arm was 5 months in both cohorts, DTC and MTC. Using a one-sided alpha of 10%, to detect a 50% reduction of risk of progression or death, i.e., HR = 0.5, with 85% power, 50 events (progression or death) were required. Assuming an accrual rate of five patients with DTC per month and accounting for 5% dropout, 50 events were expected to be observed in 75 patients entering the study over a period of 21 months and followed for an additional 7.3 months. Assuming an accrual rate of 3.3 patients with MTC per month and adding an extra 5% patients to consider patient lost to follow up, a total of 67 eligible patients were needed within 27 months of accrual. In addition, 7.4 months of follow-up time was required to have the required number of events.

Time-to-event endpoints are estimated by the Kaplan–Meier technique; confidence intervals (CIs) of medians of key endpoints are calculated using the reflected CI method. In accordance with the one-sided 10% alpha, one-sided 90% or equivalently two-sided 80% CI will be reported for these endpoints; 95% CI will be used for other descriptive measures. Cox regression using a score test, adjusted for stratification factors, was used for comparisons between treatment arms using a one-sided 10% significance level.

## Results

### Patients

From 18 June 2014 to 31 January 2018, a total of 101 patients from 21 European institutions across nine countries (Belgium, Denmark, France, Germany, Italy, The Netherlands, Poland, Spain, and the UK) were randomized in the study.

#### RAIR DTC cohort

**Figure 1:**
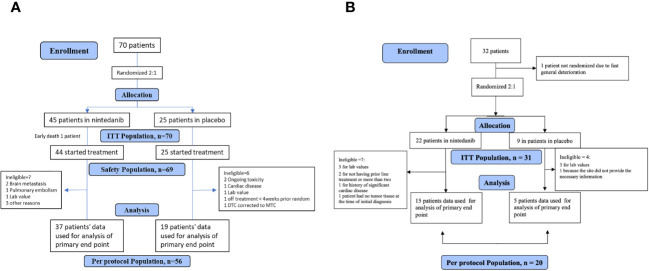
**(A)** CONSORT flowchart: RAIR DTC cohort. **(B)** CONSORT flowchart: MTC cohort.

Seventy patients diagnosed with RAIR DTC who had progressed after one (76%) or two lines (24%) of previous systemic therapy were randomized (45 to nintedanib and 25 to placebo), out of whom, 56 were included in the PP analysis (37 in the nintedanib arm and 19 in the placebo arm; see **Figure 1A**).

There are a total of seven patients allocated to the treatment arm who are deemed ineligible, with reasons including two cases of brain metastasis, one case of pulmonary embolism, one case related to lab values, and three cases for other reasons. There are six patients allocated to the placebo arm who are deemed ineligible, with reasons including two cases of ongoing toxicity, one case of cardiac disease, one case related to lab values, one case of being off treatment for less than 4 weeks prior to randomization, and one case of a diagnosis corrected from DTC to MTC.

The median age of patients in the nintedanib arm was 66 years (range, 43–79.5) and in the placebo arm was 64 years (range, 47.–81.5) (refer to **Table 1**). Approximately 56% of patients in both treatment arms were women. All patients presented with metastatic disease. Slightly over half of the patients (56%) had a performance status 1 (58% in the nintedanib arm vs. 52% in the placebo arm). The majority (93%) had undergone total thyroidectomy (91% in the nintedanib arm vs. 96% in the placebo arm) (see **Table 1**).

**Table 1:** Baseline demography and prior treatments in RAIR DTC/MTC.

	DTC cohort			MTC cohort		
	Placebo	Nintedanib	Total	Placebo	Nintedanib	Total
	(n = 25)	(n = 45)	(n = 70)	(n = 9)	(n = 22)	(n = 31)
Disease stage						
Metastatic	100%	100%	100%	100%	95.5%	96.8%
Sex						
Male	44%	44.4%	44.3%	77.8%	72.7%	74.2%
Female	56%	55.6%	55.7%	22.2%	27.3%	25.8%
Age category						
≤ 65	52%	44.4%	47.1%	77.8%	72.7%	74.2%
>65	48%	55.6%	52.9%	22.2%	27.3%	25.8%
Range	47.5–81.5	43.1–79.5	43.1–81.5	39.8–73.0	32.7–74.5	32.7–74.5
WHO status						
0	48%	42.2%	44.3%	66.7%	50%	54.8%
1	52%	57.8%	55.7%	33.3%	50%	45.2%
Thyroid surgery						
No				0%	13.6%	9.7%
Lobectomy	4%	2.2%	2.9%			
Sub-total thyroidectomy	0%	6.7%	4.3%			
Total thyroidectomy	96%	91.1%	92.9%	100%	86.4%	90.3%
Node dissection						
No	32%	33.3%	32.9%	22.2%	27.3%	25.8%
Yes	56%	48.9%	51.4%	77.8%	68.2%	71%
Missing	12%	17.8%	15.7%	0%	4.5%	3.2%
Radioiodine (RAI) therapy						
No				100%	90.9%	93.5%
Yes	100%	100%	100%	0%	9.1%	6.5%
External radiotherapy						
No	36%	40%	38.6%	44.4%	36.4%	38.7%
Yes	64%	57.8%	60%	55.6%	63.6%	31.3%
Missing	0%	2.2%	1.4%			
Systemic anticancer therapy						
Yes, received one line	76%	75.6%	75.7%	77.8%	63.6%	67.7%
Yes, received two lines	24%	24.4%	24.3%	22.2%	36.4%	32.3%
**Specify first line systemic anticancer therapy (n)**	25	45	70	25	45	70
Targeted agent, TKI	96%	97.8%	97.1%	100%	90.9%	93.5%
Cytotoxic chemotherapy	4%	2.2%	2.9%	0%	9.1%	6.5%
**Specify second-line systemic anticancer therapy (n)**	6	11	17	6	11	17
Targeted agent, TKI	83.3%	100%	94.1%	100%	87.5%	90%
Cytotoxic chemotherapy	16.7%	0%	5.9%			
Other				0%	12.5%	10%
Disease Stage						
Metastatic	25	45	70	9	21	30
Sex						
Male	11	20	31	7	16	23
Female	14	25	39	2	6	8
Age category						
≤ 65	13	20	33	7	16	23
>65	12	25	37	2	6	8
Range	47.5 - 81.5	43.1 – 79.5	43.1 - 81.5	39.8 – 73.0	32.7 – 74.5	32.7 – 74.5
WHO status						
0	12	19	31	6	11	17
1	13	26	39	3	11	14
Thyroid surgery						
No				0	3	3
Lobectomy	1	1	2			
Sub-total thyroidectomy	0	3	3			
Total thyroidectomy	24	41	65	9	19	28
Node dissection						
No	8	15	23	2	6	8
Yes	14	22	36	7	15	22
Missing	3	8	11	0	1	1
Radioiodine (RAI) therapy						
No				9	20	29
Yes	25	45	70	0	2	2
External radiotherapy						
No	9	18	27	4	8	12
Yes	16	26	42	5	14	19
Missing	0	1	1			
Systemic anticancer therapy						
Yes, received 1 line	19	34	53	7	14	21
Yes, received 2 lines	6	11	17	2	8	10
**Specify first line systemic anticancer therapy (n)**	25	45	70	25	45	70
Targeted agent, TKI	24	44	68	9	20	29
Cytotoxic chemotherapy	1	1	2	0	2	2
**Specify second line systemic anticancer therapy (n)**	6	11	17	6	11	17
Targeted agent, TKI	5	11	16	2	7	9
Cytotoxic chemotherapy	1	0	1			
Other				0	1	1

Prior treatments for thyroid cancer included sorafenib in 37 patients (24 in the nintedanib arm and 13 in the placebo arm), pazopanib in 12 patients (seven in the nintedanib arm and five in the placebo arm), lenvatinib in 10 patients (eight in the nintedanib arm and two in the placebo arm), and vandetanib in 10 patients (five in the nintedanib arm and five in the placebo arm). One patient randomized to the nintedanib arm did not initiate treatment due to early death.

#### MTC cohort

Thirty-one (31 out of the 67 planned) patients diagnosed with MTC who had progressed after one (68%) or two (32%) lines of previous systemic therapy were randomized (22 to the nintedanib arm and nine to the placebo arm; see **Figure 1B**). The MTC cohort was closed early due to slow accrual. There are seven patients allocated to the treatment arm who are deemed ineligible, with reasons including two cases of not received prior line treatment, one case of not tumor tissue at the time of diagnosis, three cases related to lab values, and one case of severe cardiac disease. There are four patients allocated to the placebo arm who are deemed ineligible, with reasons including three cases related to lab values and one case due to a lack of information regarding the disease.

The median age of patients in the nintedanib arm was 55 years (range, 33–74.5) and in the placebo arm was 60 years (range, 40–73) (refer to **Table 1**).

Both arms had a higher percentage of male patients compared to female patients (72.7% in the nintedanib arm and 77.8% in the placebo arm). All patients but one (96.8%) had metastatic disease; total thyroidectomy was performed in 28 patients (90.3%) with nodal dissection in 22 (71.0%) (**Table 1**). Most patients (67.7%) had received one prior line of systemic therapy, whereas 32.3% had received a second line. Among the 29 patients who received targeted treatment, all were treated with vandetanib (20 with nintedanib and nine with placebo). Only two patients received cytotoxic chemotherapy and/or another investigational drug as first-line treatment (refer to **Table 1**)

### Duration of treatment

#### RAIR DTC cohort

At the time of data cutoff (5 October 2017), the median follow-up in the ITT population was 26.3 months (Interquartile Range (IQR), 14.4–29.2) in the nintedanib arm and 19.8 months (IQR, 13.6–26.9) in the placebo arm. Five patients continued receiving blinded treatment (four were receiving nintedanib and one was receiving placebo) at the time of data cutoff. Among 24 eligible patients who received placebo and had tumor progression, 14 patients elected to receive open-label nintedanib, two were treated by other tyrosine kinase inhibitors (TKIs), and eight received best supportive care.

Based on 69 patients who started treatment, the median duration of treatment was 20 (95% CI, 12–28) weeks in the nintedanib arm and 9 weeks (95% CI, 8–16) in the placebo arm (*P* = 0.580). The median relative dose intensity was 99.9% (range, 53.5–112.5) and 100% (range, 67.4–106.1) in the nintedanib and placebo arm, respectively.

#### MTC cohort:

At the time of data cutoff (28 August 2019), the median follow-up in the ITT population was 48.4 months (IQR, 20.3–50.6) in the nintedanib group and 19.7 months (IQR, 11.1–48.2) in the placebo group. Median treatment duration was 19 weeks (95% CI, 8–30) for the nintedanib arm against 16 weeks (95% CI, 8–24) in the placebo arm. Four patients (12.9%) in the nintedanib arm received treatment for a period exceeding 35 weeks. The median relative dose intensity (RDI) was 91.3% (range, 45.8–100.0) for nintedanib and 100% (range, 76.4–101.1) for placebo.

### Efficacy

#### RAIR DTC cohort

**Figure 2:**
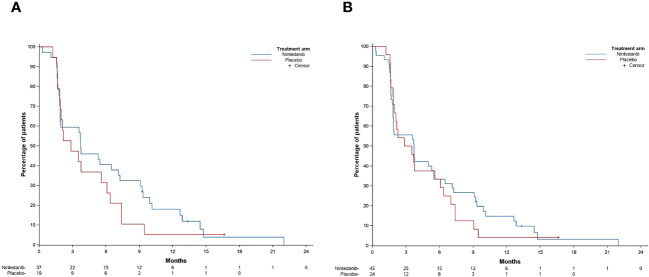
Progression-free survival (PFS) in the RAIR DTC per-protocol population **(A)** and in the intention-to-treat (ITT) population **(B)**.

Based on the PP population, among the 56 randomized patients, there were 53 events observed for the primary endpoint. Median PFS was 3.7 months (80% CI, 1.9–6.5) in the nintedanib arm and 2.9 months (80% CI, 2.0–5.6) in the placebo arm (**Figure 2**). The p-value was 0.095 with a HR of 0.65 (80% CI, 0.42–0.99), demonstrating a significantly prolonged PFS of nintedanib compared to placebo. Analysis in the ITT population (70 patients) showed a median PFS of 3.7 months (80% CI, 1.9–5.0) in the nintedanib arm and 2.9 months (80% CI, 2.1–3.8) in the placebo arm (p-value = 0.126; HR, 0.73; 80% CI, 0.51–1.04), which was not significant.

Among patients from the PP population the best OR was stable disease (SD) in 22 patients (60%) in the nintedanib and nine patients (47%) in the placebo arm. No objective response was observed.

Twenty-two out of the 56 patients died, 15 in the nintedanib arm and seven in the placebo arm. Tumor progression was the cause of death for 19 patients. Median OS was 29.6 months (80% CI, 15.1–not reached) in the nintedanib arm and not reached in the placebo arm. The 1-year survival rate was 77.5% (80% CI, 66.9–85.1) in the nintedanib arm and 73.7% (80% CI, 66.9–85.1) in the placebo arm (HR = 1.00; 80% CI, 0.54–1.84; *P* = 0.5).

Median OS in the ITT population was 20.4 months (80% CI, 15.2–29.6) in the nintedanib arm and 20.6 (80% CI, 13.7–not reached) in the placebo arm. The 1-year survival rate was 70.3% (80% CI, 60.5–78.2) in the nintedanib arm and 64.0% (80% CI, 50.3–74.8) in the placebo arm (HR = 0.93; 80% CI, 0.58–1.50; *P* = 0.42).

#### MTC cohort

As the MTC cohort was stopped due to slow accrual, no inferential tests were performed, and, therefore, HR for OS and PFS are provided with 95% confidence interval only.

A PFS event was observed for all 20 patients of the PP population. Median PFS was 7.0 months (80% CI, 1.9–8.7) in the nintedanib arm and 3.9 months (80% CI, 3.0–5.5) in the placebo arm. The corresponding (unadjusted) HR for the PFS of nintedanib relative to placebo was 0.49 (95% CI, 0.16–1.53). Median OS was 16.4 months (80% CI, 12.1–24.9) in the nintedanib arm and 12.3 months (80% CI, 7.1–not reached) in the placebo arm (HR = 0.88; 95% CI, 0.24–3.21).

No objective response has been observed; patients had either stable (13/20, 65%) or PD (6/20, 30%). In one patient, data on activity were not evaluable.

### Safety

**Supplementary Table 1** displays AEs observed during the blinded period in the combined safety population of the two cohorts, consisting of 66 patients in the nintedanib arm and 34 patients in the placebo arm. Among these, 62 patients (93%) in the nintedanib arm and 33 patients (97.1%) in the placebo arm experienced at least one AE. Grade 3 or higher AEs were reported in 35 patients (53%) in the nintedanib arm and 12 patients (35%) in the placebo arm. The most common grade 3 or higher AEs, occurring in at least 5% of patients, were diarrhea (12.1%), gamma-glutamyl transferase increase (10.6%), anorexia (9.1%), nausea (6.1%), and hypertension (6.1%) in the nintedanib arm (see **Supplementary Table 2**).

## Discussion

This study investigated the efficacy and safety of nintedanib in patients with locally advanced or metastatic progressive RAIR DTC and MTC who had previously systemically received one or two lines of MKIs. At the start of the trial in 2014, there were no approved second-line or third-line treatments for advanced thyroid cancers, hence the use of a placebo as the control arm.

Nintedanib demonstrated a significant PFS advantage over placebo in the PP analysis of the RAIR DTC cohort. However, this advantage was not observed in the ITT population. Despite that the PP population is the population of interest for the primary endpoint, the impact on PFS is minimal and not clinically relevant, especially when compared to the efficacy demonstrated in other studies involving similar or earlier disease populations. For instance, sorafenib showed a PFS increase of 5 months (HR = 0.59) (1) compared to placebo, lenvatinib showed an increase of almost 14.7 months (HR = 0.21) (2), and cabozantinib showed an increase of 9.1 months (3). This latter was recently approved by the FDA in 2021 and by the European Medicines Agency (EMA) in 2022 as a second-line treatment for RAIR DTC after lenvatinib and/or sorafenib, showing a significant improvement in PFS compared to placebo (HR = 0.22; 96% CI, 0.15–0.32) (3).

Various second-line treatment options are currently available, and personalized agents may be considered for oncogene-addicted tumors (e.g., Neurotrophic Tyrosine Receptor Kinase (NTRK), Anaplastic Lymphoma Kinase (ALK), Rearranged During Transfection (RET), and B-Raf Proto-Oncogene, Serine/Threonine Kinase (BRAF)) (4–9), even in the absence of randomized trials. NTRK inhibitors (larotrectinib and entrectinib) (5, 6) have received agnostic approval from both the EMA and FDA, thanks to their activity. Selpercatinib has been approved as a first-line treatment by the FDA and as a second-line treatment by the EMA for RAIR DTC in RET-fusion–positive tumors and as a first-line treatment for MTC in RET-mutated tumors by both agencies (4). Pralsetinib, another selective RET inhibitor drug, is approved as a first-line treatment by the FDA for both RAIR DTC (RET-fusion positive) and MTC (RET-mutation) (8, 9). For cases with the BRAFV600E mutation, BRAF and MEK inhibitors (dabrafenib and trametinib) (10) have the FDA approval for agnostic use. The investigation of the BRAF mutation was not conducted, nor was it deemed necessary as per the protocol eligibilities.

In the MTC cohort, recruitment was stopped prematurely due to slow enrolment resulting in a final PP population of 20 patients. Consequently, the modified statistical analysis plan did not reach the targeted statistical power for the final analysis. Whether the median PFS of placebo arm was superimposable to the median PFS observed in the placebo group of the EXAM trial (4.0 months), median PFS obtained with nintedanib is far from the 11.2 months observed with cabozantinib (11) or with the “not estimable” value reported with pralsetinib (8) or selpercatinib (9) in the RET-mutated MTC. Molecular analyses were not conducted, nor were they deemed necessary per protocol. Even if somatic tumor profile is currently included in the work-up of patients with DTC and MTC with relapsed and/or metastatic disease, when the trial started, it was not required.

**Figure 3:**
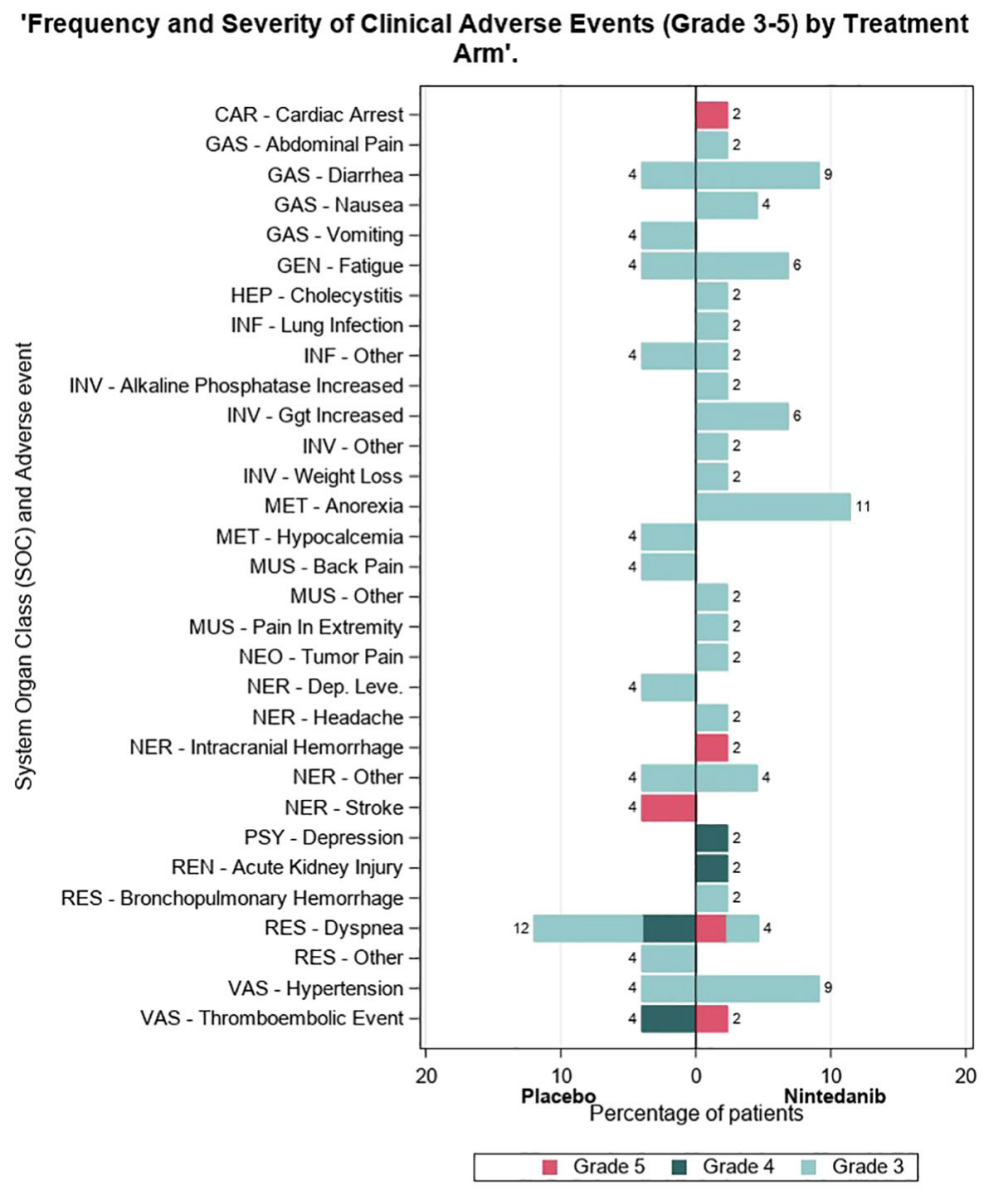
Frequency of clinical adverse events of the nintedanib-treated group compared to the placebo group during randomized treatment in both cohorts.

The hypothesis of cross-resistance with prior treatments may be considered, as more than half of both patient populations were treated as second line and approximately one-fourth as third line. However, one must recognize that other MKIs like cabozantinib and lenvatinib have shown RECIST 1.1 partial response rates ranging from 11% to 52.3% in RAIR DTC (2, 3) and 23% with cabozantinib in MTC (11), even when administered in second- or third-line settings. The safety profile of nintedanib aligns with the known class effects. AEs were more frequently reported in the nintedanib-treated group compared to the placebo group during randomized treatment in both cohorts (see **Figure 3**). However, toxicity levels were lower than those reported for lenvatinib, with only 7% of patients discontinuing nintedanib due to AE compared to 14% for lenvatinib and 19% for sorafenib (1, 2). Similarly, dose interruptions due to toxicity were less common under nintedanib at 8%, compared to 82% for lenvatinib and 66% for sorafenib.

The study faced several limitations. A significant number of patients were randomized without strict adherence to all eligibility criteria, although all patients received treatment as eligibility criteria were retrospectively verified. Certain conditions such as brain metastases and recent serious cardiovascular events (< 6 months) may have led to a biased patient selection, potentially reducing the probability of success. Important data on thyroglobulin, thyroid-stimulating hormone (TSH), calcitonin, carcinoembryonic antigen (CEA) levels, and molecular profiles were not collected. MKIs are known to elevate TSH levels, necessitating adjustments in thyroid hormone replacement dosages (1, 2). Persistent high TSH levels could promote tumor growth in DTC and potentially mask the efficacy of nintedanib. However, given the lack of tumor response in any patient, nintedanib should be deemed ineffective in patients with refractory advanced iodine-refractory DTC and MTC who have previous received other TKIs.

In conclusion, although nintedanib showed a PFS extension compared to placebo in patients with refractory advanced iodine-refractory DTC and MTC who had prior TKI treatment in the PP population, this study did not indicate a significant clinical improvement in PFS. The observed effect was not deemed substantial to justify further development of the compound for this indication.

## Data availability statement

The raw data supporting the conclusions of this article will be made available by the authors, without undue reservation.

## Ethics statement

Ethics committee approved the study in each site involved in the research. The studies were conducted in accordance with the local legislation and institutional requirements. The participants provided their written informed consent to participate in this study.

## Author contributions

SLe: Writing – review & editing, Writing – original draft, Methodology. EK: Writing – review & editing, Conceptualization, Data curation. SLi: Data curation, Writing – review & editing, Methodology. PS: Writing – review & editing, Conceptualization, Supervision. YG: Supervision, Writing – review & editing, Methodology. PR: Supervision, Writing – review & editing, Validation. BJ: Writing – review & editing, Conceptualization, Data curation. DS: Writing – review & editing, Supervision. SZ: Supervision, Writing – review & editing. JC: Supervision, Writing – review & editing, Methodology. LB: Supervision, Writing – review & editing. CD: Writing – review & editing, Data curation. YL: Writing – review & editing, Methodology, Supervision. SB: Methodology, Supervision, Writing – review & editing. FC: Methodology, Writing – review & editing, Investigation. MD: Writing – review & editing, Supervision. TL: Supervision, Writing – review & editing. WS: Writing – review & editing, Methodology. MS: Methodology, Writing – review & editing. DLL: Writing – review & editing, Conceptualization, Supervision, Visualization, Writing – original draft. KN: Methodology, Writing – review & editing.

## Supplementary material

Supplementary Table 1 Adverse events observed during blinded treatment (Grade ≥ 3) (NCI-CTCAE version 4.0) (Safety population).

Supplementary Table 2 All adverse events, by treatment.
