# Supplementary material for: Safety and efficacy of nintedanib as second-line therapy for patients with differentiated or medullary thyroid cancer progressing after first-line therapy. A randomized phase II study of the EORTC Endocrine Task Force (protocol 1209-EnTF)
Source: Front Endocrinol (Lausanne). 2024 Jun 27;15:1403687. doi: 10.3389/fendo.2024.1403687 (PMC11250516; doi:10.3389/fendo.2024.1403687)
Supplement: Supplementary file 1 [file Presentation_1.pptx]

## Slide 1
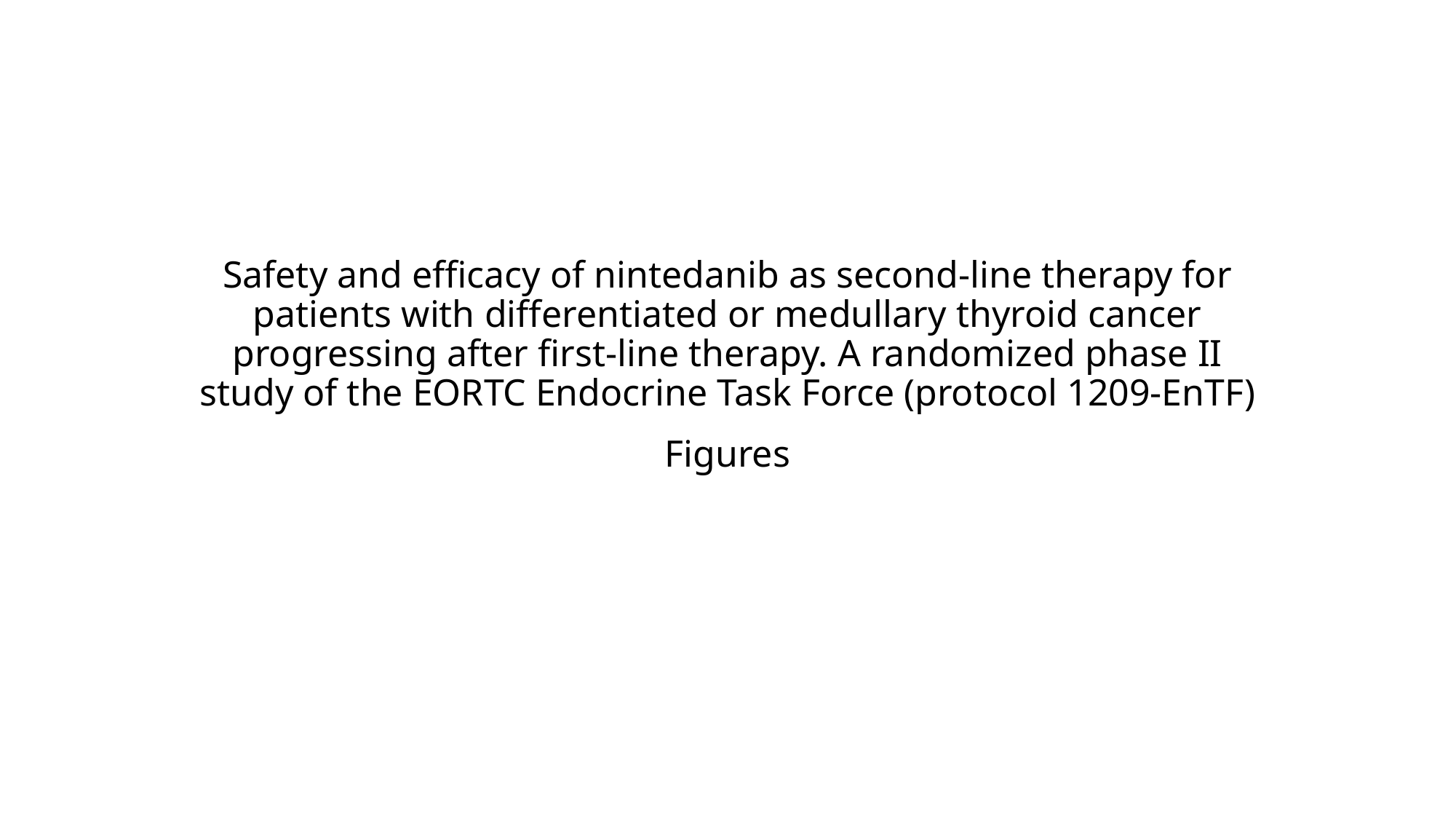

# Safety and efficacy of nintedanib as second-line therapy for patients with differentiated or medullary thyroid cancer progressing after first-line therapy. A randomized phase II study of the EORTC Endocrine Task Force (protocol 1209-EnTF)
Figures

## Slide 2
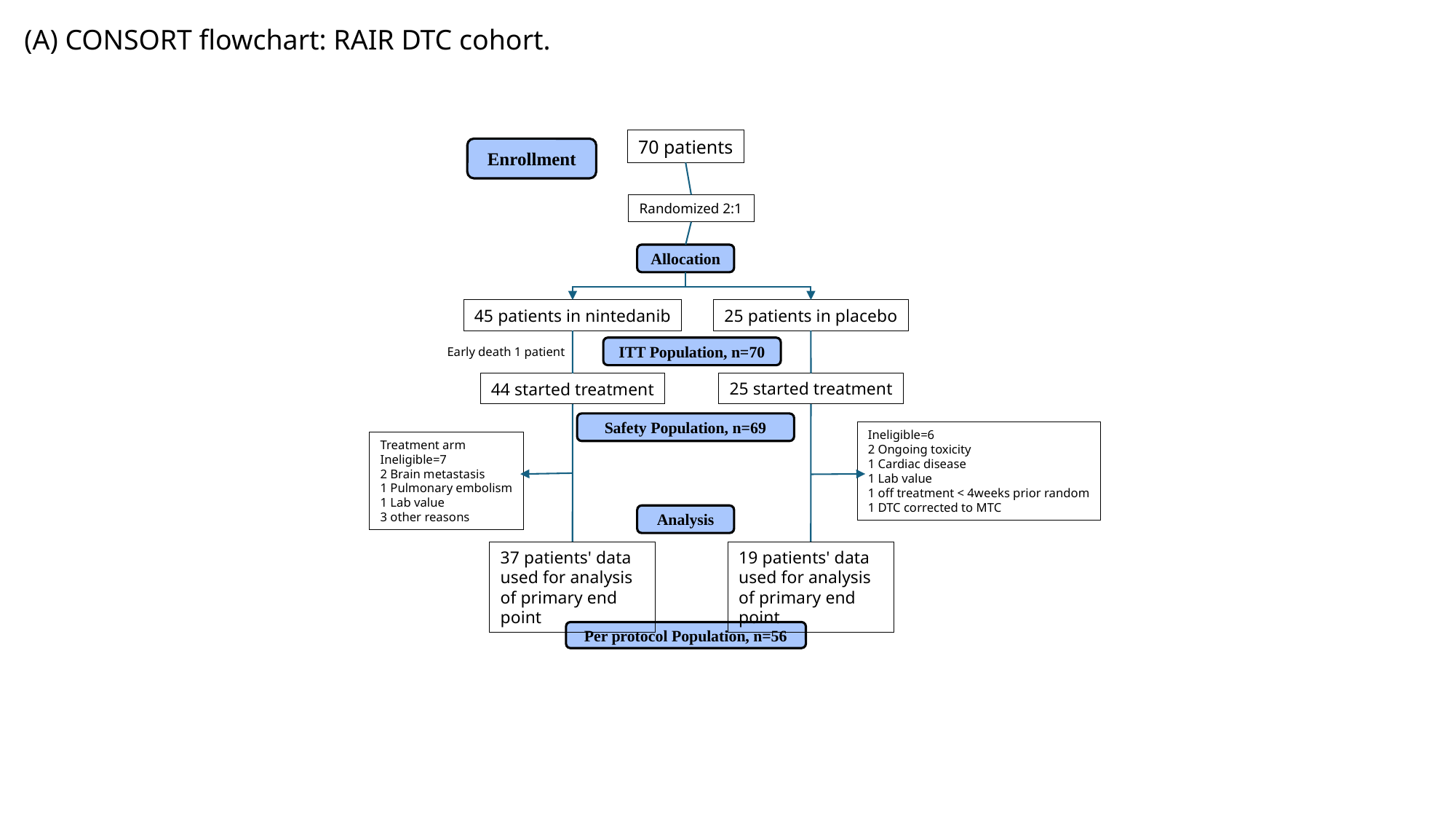

(A) CONSORT flowchart: RAIR DTC cohort.
70 patients
Enrollment
Randomized 2:1
Allocation
45 patients in nintedanib
25 patients in placebo
ITT Population, n=70
Early death 1 patient
25 started treatment
44 started treatment
Safety Population, n=69
Ineligible=6
2 Ongoing toxicity
1 Cardiac disease
1 Lab value
1 off treatment < 4weeks prior random
1 DTC corrected to MTC
Treatment arm
Ineligible=7
2 Brain metastasis
1 Pulmonary embolism
1 Lab value
3 other reasons
Analysis
37 patients' data used for analysis of primary end point
19 patients' data used for analysis of primary end point
Per protocol Population, n=56

## Slide 3
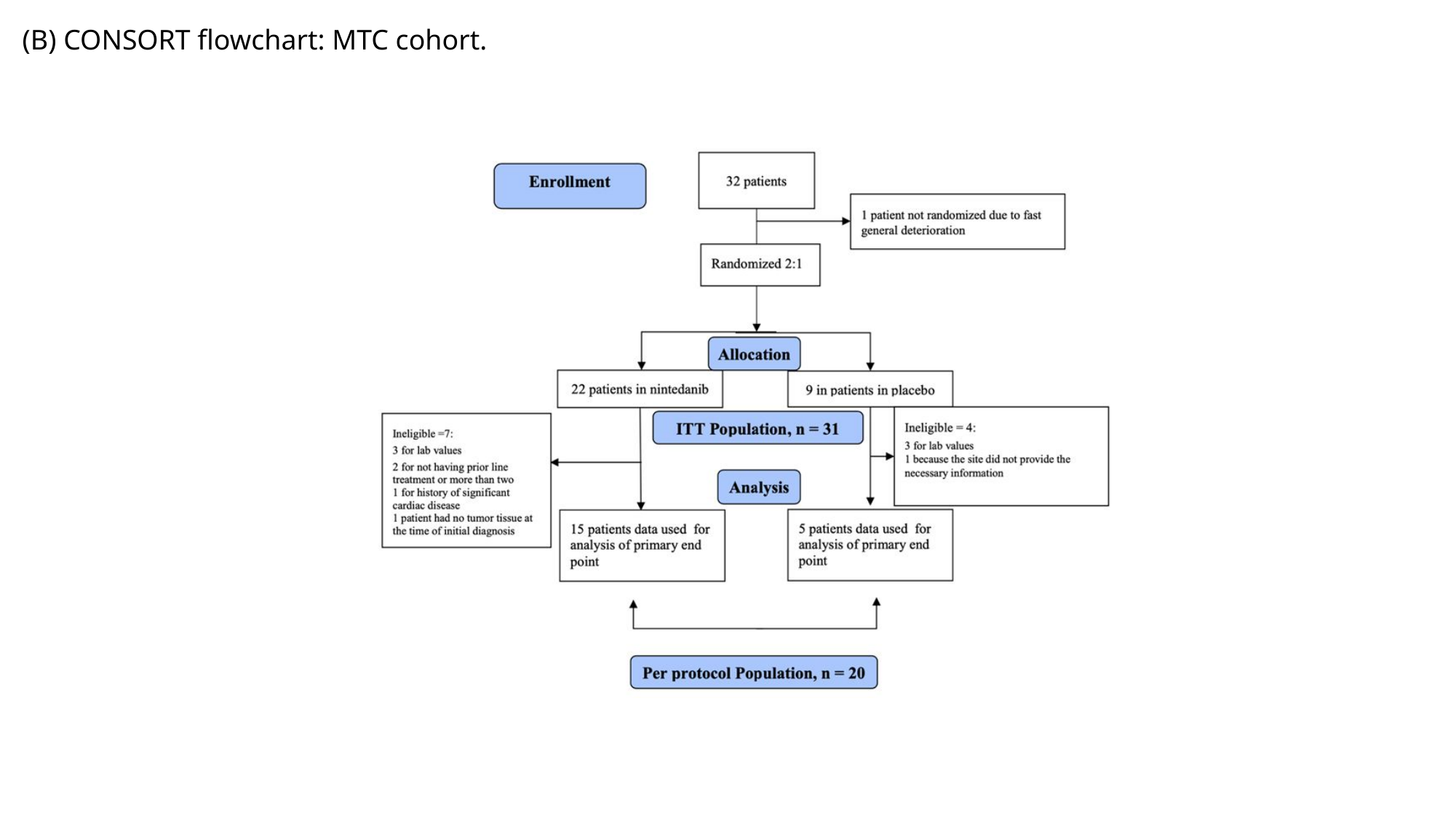

(B) CONSORT flowchart: MTC cohort.

## Slide 4
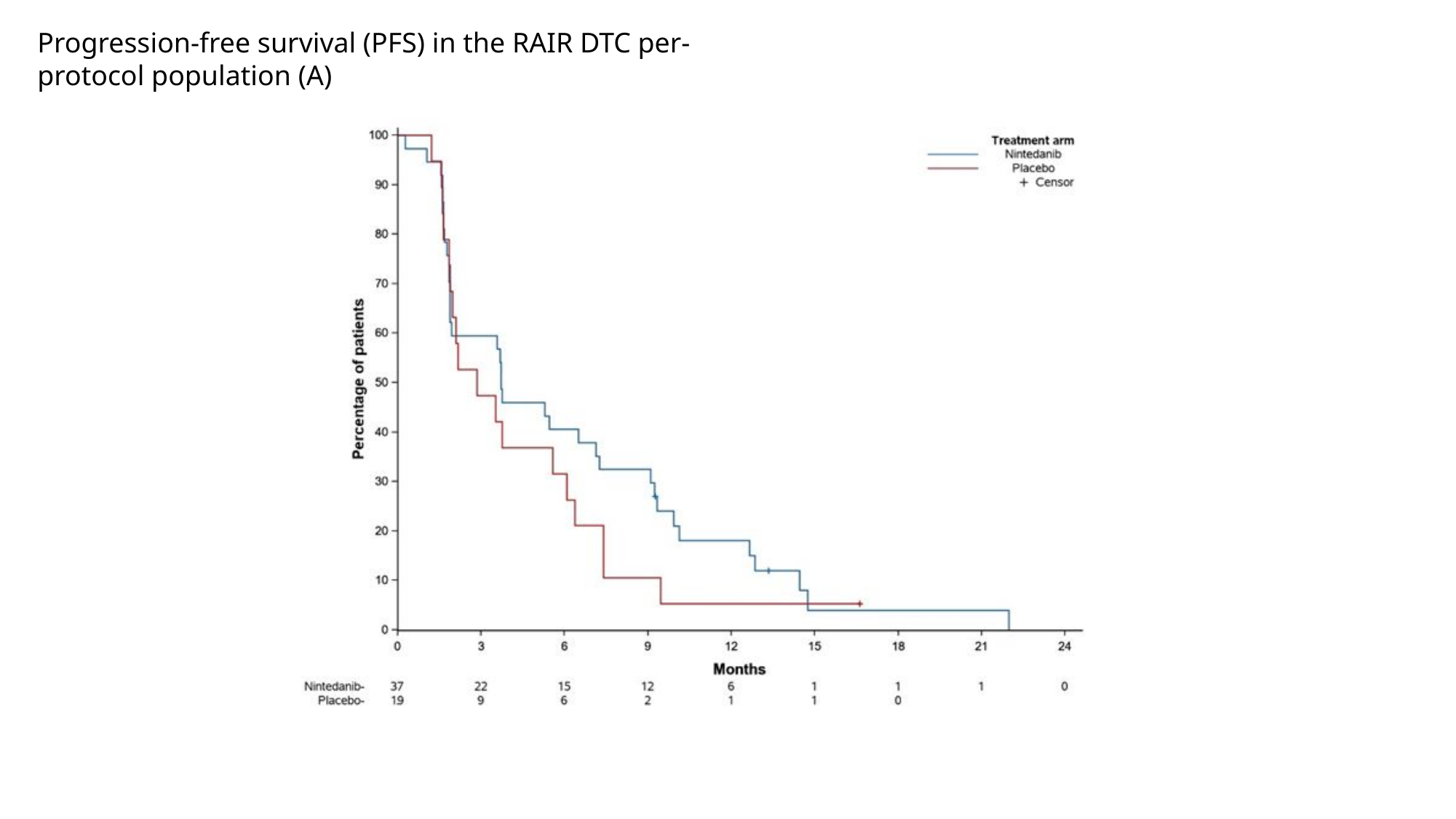

Progression-free survival (PFS) in the RAIR DTC per-protocol population (A)

## Slide 5
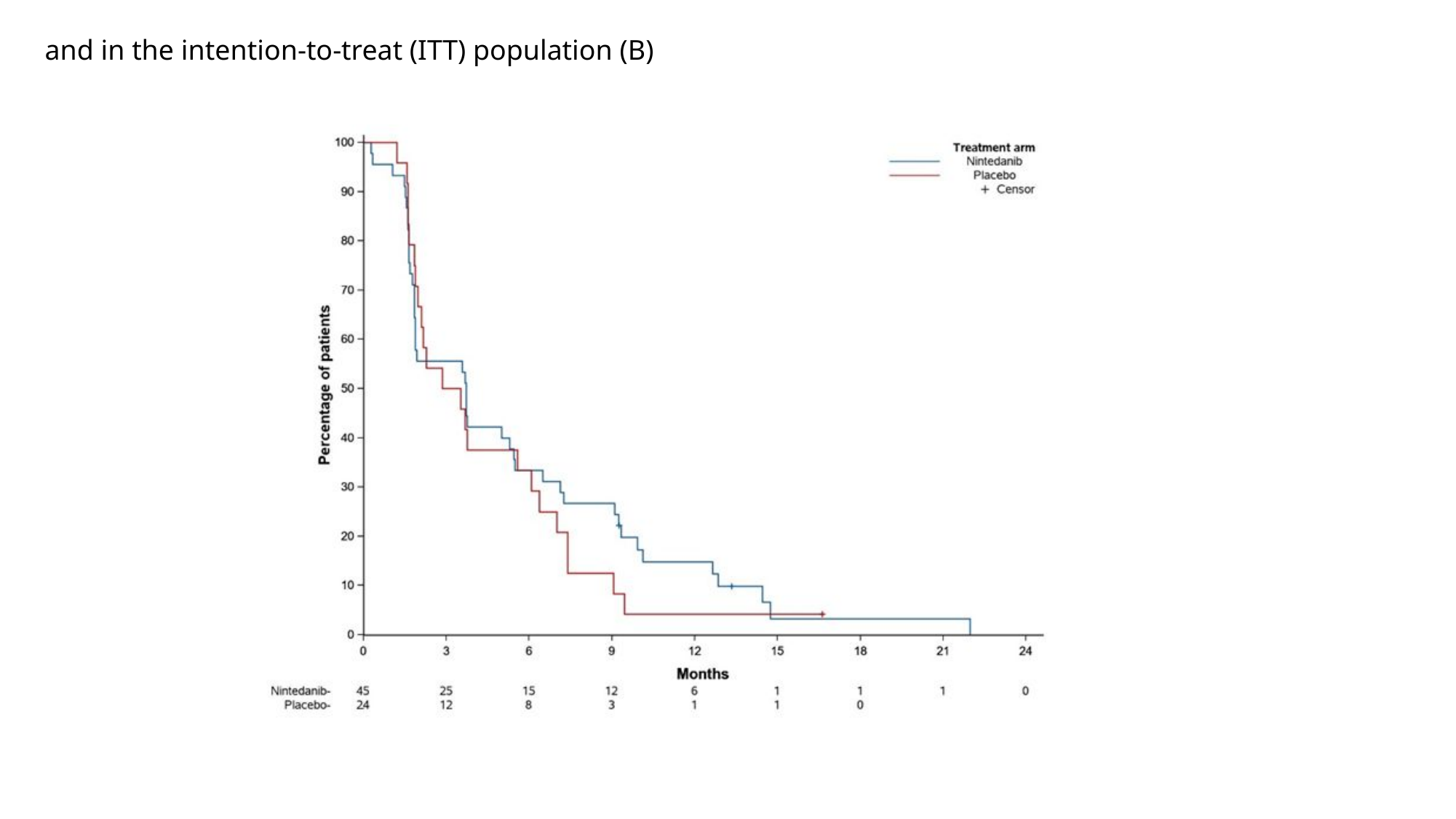

and in the intention-to-treat (ITT) population (B)

## Slide 6
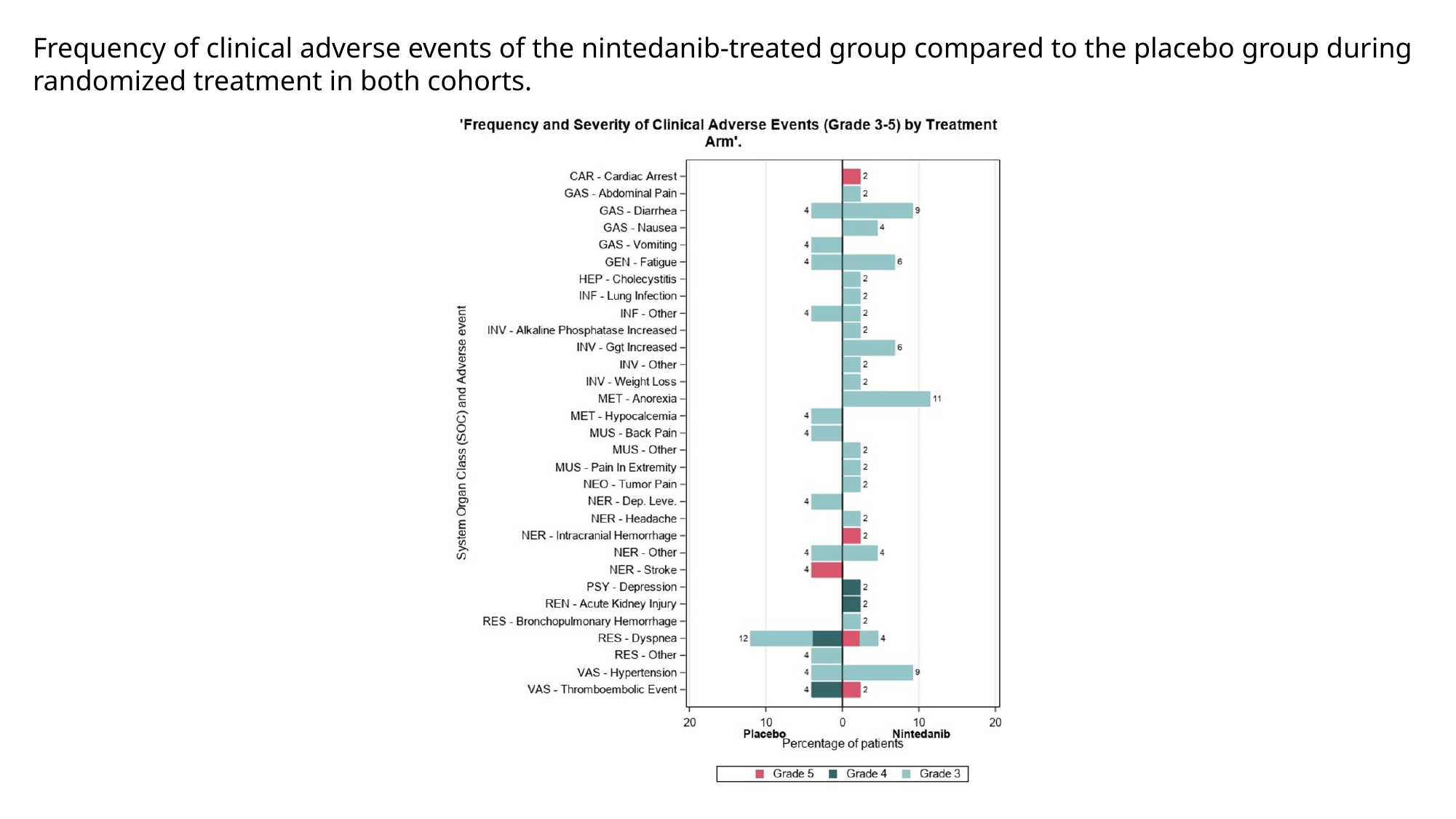

Frequency of clinical adverse events of the nintedanib-treated group compared to the placebo group during randomized treatment in both cohorts.
