# Supplementary material for: Safety and efficacy of nintedanib as second-line therapy for patients with differentiated or medullary thyroid cancer progressing after first-line therapy. A randomized phase II study of the EORTC Endocrine Task Force (protocol 1209-EnTF)
Source: Front Endocrinol (Lausanne). 2024 Jun 27;15:1403687. doi: 10.3389/fendo.2024.1403687 (PMC11250516; doi:10.3389/fendo.2024.1403687)
Supplement: Supplementary Table 1 — Adverse events observed during blinded treatment (Grade ≥ 3) (NCI-CTCAE version 4.0) (Safety population). [file Table_1.docx]

Safety and efficacy of nintedanib as second-line therapy for patients with differentiated or medullary thyroid cancer progressing after first-line therapy. A randomized phase II study of the EORTC Endocrine Task Force (protocol 1209-EnTF).

Supplemental table 1: Adverse events observed during blinded treatment (Grade ≥ 3) (NCI-CTCAE version 4.0) (Safety population)

|  | **Placebo (Safety pop:N=34)** | | | | | | | **Nintedanib (Safety pop:N=66)** | | | | | | |
| --- | --- | --- | --- | --- | --- | --- | --- | --- | --- | --- | --- | --- | --- | --- |
| **System Organ Class + Preferred term** | **Grade  1  N (%)** | **Grade  2  N (%)** | **Grade  3  N (%)** | **Grade  4  N (%)** | **Grade  5  N (%)** | **Grade  ≥3  N (%)** | **Grade  ≥1  N (%)** | **Grade  1  N (%)** | **Grade  2  N (%)** | **Grade  3  N (%)** | **Grade  4  N (%)** | **Grade  5  N (%)** | **Grade  ≥3  N (%)** | **Grade  ≥1  N (%)** |
| PATIENTS' WORST GRADE | 10 (29.4) | 11 (32.4) | 8 (23.5) | 2 (5.9) | 2 (5.9) | 12 (35.3) | 33 (97.1) | 10 (15.2) | 17 (25.8) | 30 (45.5) | 2 (3.0) | 3 (4.5) | 35 (53.0) | 62 (93.9) |
| BLOOD AND LYMPHATIC SYSTEM DISORDERS |  |  |  |  |  |  |  |  |  |  |  |  |  |  |
| Other AE |  |  |  |  |  |  |  | 1 (1.5) |  |  |  |  |  | 1 (1.5) |
| CARDIAC DISORDERS |  |  |  |  |  |  |  |  |  |  |  |  |  |  |
| Atrial Fibrillation |  | 2 (5.9) |  |  |  |  | 2 (5.9) |  | 2 (3) |  |  |  |  | 2 (3) |
| Cardiac Arrest |  |  |  |  |  |  |  |  |  |  |  | 1 (1.5) | 1 (1.5) | 1 (1.5) |
| Heart Failure |  |  |  |  |  |  |  |  | 1 (1.5) |  |  |  |  | 1 (1.5) |
| Palpitations | 1 (2.9) |  |  |  |  |  | 1 (2.9) |  |  |  |  |  |  |  |
| Pericardial Effusion |  |  |  |  |  |  |  |  |  | 1 (1.5) |  |  | 1 (1.5) | 1 (1.5) |
| ENDOCRINE DISORDERS |  |  |  |  |  |  |  |  |  |  |  |  |  |  |
| Hyperthyroidism |  | 1 (2.9) |  |  |  |  | 1 (2.9) |  |  |  |  |  |  |  |
| EYE DISORDERS |  |  |  |  |  |  |  |  |  |  |  |  |  |  |
| Blurred Vision | 1 (2.9) |  |  |  |  |  | 1 (2.9) |  |  |  |  |  |  |  |
| Other AE | 1 (2.9) |  |  |  |  |  | 1 (2.9) |  |  |  |  |  |  |  |
| GASTROINTESTINAL DISORDERS |  |  |  |  |  |  |  |  |  |  |  |  |  |  |
| Abdominal Pain | 1 (2.9) |  |  |  |  |  | 1 (2.9) | 3 (4.5) | 5 (7.6) | 2 (3) |  |  | 2 (3) | 10 (15.2) |
| Anal Hemorrhage |  |  |  |  |  |  |  | 1 (1.5) |  |  |  |  |  | 1 (1.5) |
| Colitis |  | 1 (2.9) |  |  |  |  | 1 (2.9) |  |  |  |  |  |  |  |
| Colonic Perforation |  | 1 (2.9) |  |  |  |  | 1 (2.9) |  |  |  |  |  |  |  |
| Constipation |  |  |  |  |  |  |  | 4 (6.1) | 2 (3) |  |  |  |  | 6 (9.1) |
| Diarrhea | 4 (11.8) | 1 (2.9) | 1 (2.9) |  |  | 1 (2.9) | 6 (17.6) | 15 (22.7) | 11 (16.7) | 8 (12.1) |  |  | 8 (12.1) | 34 (51.5) |
| Dry Mouth | 3 (8.8) |  |  |  |  |  | 3 (8.8) |  | 1 (1.5) |  |  |  |  | 1 (1.5) |
| Dysphagia |  |  |  |  |  |  |  | 2 (3) |  |  |  |  |  | 2 (3) |
| Enterocolitis |  |  |  |  |  |  |  |  | 1 (1.5) |  |  |  |  | 1 (1.5) |
| Gastritis |  |  |  |  |  |  |  | 1 (1.5) |  |  |  |  |  | 1 (1.5) |
| Gastroesophageal Reflux Disease |  | 1 (2.9) |  |  |  |  | 1 (2.9) | 1 (1.5) |  |  |  |  |  | 1 (1.5) |
| Gastrointestinal Pain |  |  |  |  |  |  |  | 1 (1.5) |  |  |  |  |  | 1 (1.5) |
| Mucositis Oral | 1 (2.9) |  |  |  |  |  | 1 (2.9) | 1 (1.5) | 1 (1.5) |  |  |  |  | 2 (3) |
| Nausea | 2 (5.9) | 1 (2.9) |  |  |  |  | 3 (8.8) | 14 (21.2) | 5 (7.6) | 4 (6.1) |  |  | 4 (6.1) | 23 (34.8) |
| Oral Pain |  |  |  |  |  |  |  | 1 (1.5) |  |  |  |  |  | 1 (1.5) |
| Stomach Pain |  |  |  |  |  |  |  | 3 (4.5) | 1 (1.5) |  |  |  |  | 4 (6.1) |
| Vomiting | 2 (5.9) |  | 1 (2.9) |  |  | 1 (2.9) | 3 (8.8) | 9 (13.6) | 4 (6.1) | 1 (1.5) |  |  | 1 (1.5) | 14 (21.2) |
| Other AE |  |  |  |  |  |  |  | 2 (3) |  |  |  |  |  | 2 (3) |
| GENERAL DISORDERS AND ADMINISTRATION SITE CONDITIONS |  |  |  |  |  |  |  |  |  |  |  |  |  |  |
| Edema Limbs |  |  |  |  |  |  |  | 1 (1.5) |  |  |  |  |  | 1 (1.5) |
| Fatigue | 8 (23.5) | 2 (5.9) | 2 (5.9) |  |  | 2 (5.9) | 12 (35.3) | 9 (13.6) | 10 (15.2) | 4 (6.1) |  |  | 4 (6.1) | 23 (34.8) |
| Fever |  |  |  |  |  |  |  | 2 (3) | 1 (1.5) |  |  |  |  | 3 (4.5) |
| Flu Like Symptoms |  |  |  |  |  |  |  | 1 (1.5) |  |  |  |  |  | 1 (1.5) |
| Gait Disturbance |  |  |  |  |  |  |  | 1 (1.5) |  |  |  |  |  | 1 (1.5) |
| Localized Edema | 1 (2.9) |  |  |  |  |  | 1 (2.9) |  |  |  |  |  |  |  |
| Malaise |  |  |  |  |  |  |  | 1 (1.5) |  |  |  |  |  | 1 (1.5) |
| Non-Cardiac Chest Pain |  |  |  |  |  |  |  | 2 (3) |  |  |  |  |  | 2 (3) |
| Pain | 1 (2.9) |  |  |  |  |  | 1 (2.9) | 3 (4.5) | 1 (1.5) | 1 (1.5) |  |  | 1 (1.5) | 5 (7.6) |
| Other AE |  |  |  |  |  |  |  | 1 (1.5) |  |  |  |  |  | 1 (1.5) |
| HEPATOBILIARY DISORDERS |  |  |  |  |  |  |  |  |  |  |  |  |  |  |
| Cholecystitis |  |  |  |  |  |  |  |  |  | 1 (1.5) |  |  | 1 (1.5) | 1 (1.5) |
| IMMUNE SYSTEM DISORDERS |  |  |  |  |  |  |  |  |  |  |  |  |  |  |
| Allergic Reaction |  |  |  |  |  |  |  |  | 1 (1.5) |  |  |  |  | 1 (1.5) |
| Other AE |  |  |  |  |  |  |  | 1 (1.5) |  |  |  |  |  | 1 (1.5) |
| INFECTIONS AND INFESTATIONS |  |  |  |  |  |  |  |  |  |  |  |  |  |  |
| Arteritis Infective |  |  |  |  |  |  |  |  |  | 1 (1.5) |  |  | 1 (1.5) | 1 (1.5) |
| Bronchial Infection |  | 2 (5.9) |  |  |  |  | 2 (5.9) |  | 1 (1.5) |  |  |  |  | 1 (1.5) |
| Lung Infection |  |  |  |  |  |  |  |  | 1 (1.5) | 1 (1.5) |  |  | 1 (1.5) | 2 (3) |
| Sepsis |  |  |  | 1 (2.9) |  | 1 (2.9) | 1 (2.9) |  |  |  |  |  |  |  |
| Sinusitis |  |  |  |  |  |  |  |  | 1 (1.5) |  |  |  |  | 1 (1.5) |
| Tracheitis |  | 1 (2.9) |  |  |  |  | 1 (2.9) |  |  |  |  |  |  |  |
| Upper Respiratory Infection |  |  |  |  |  |  |  |  | 2 (3) |  |  |  |  | 2 (3) |
| Urinary Tract Infection |  |  |  |  |  |  |  |  | 2 (3) |  |  |  |  | 2 (3) |
| Wound Infection |  |  |  |  |  |  |  |  | 1 (1.5) |  |  |  |  | 1 (1.5) |
| Other AE |  |  | 1 (2.9) |  |  | 1 (2.9) | 1 (2.9) |  |  | 1 (1.5) |  |  | 1 (1.5) | 1 (1.5) |
| INJURY, POISONING AND PROCEDURAL COMPLICATIONS |  |  |  |  |  |  |  |  |  |  |  |  |  |  |
| Other AE | 1 (2.9) |  |  |  |  |  | 1 (2.9) |  |  |  |  |  |  |  |
| INVESTIGATIONS |  |  |  |  |  |  |  |  |  |  |  |  |  |  |
| Alkaline Phosphatase Increased |  |  |  |  |  |  |  | 2 (3) |  | 1 (1.5) |  |  | 1 (1.5) | 3 (4.5) |
| Ggt Increased | 1 (2.9) |  |  |  |  |  | 1 (2.9) |  | 1 (1.5) | 7 (10.6) |  |  | 7 (10.6) | 8 (12.1) |
| Lymphocyte Count Decreased |  |  |  |  |  |  |  |  | 1 (1.5) |  |  |  |  | 1 (1.5) |
| Serum Amylase Increased |  |  |  |  |  |  |  |  |  | 1 (1.5) |  |  | 1 (1.5) | 1 (1.5) |
| Weight Gain | 1 (2.9) | 1 (2.9) |  |  |  |  | 2 (5.9) |  |  |  |  |  |  |  |
| Weight Loss | 7 (20.6) | 1 (2.9) | 1 (2.9) |  |  | 1 (2.9) | 9 (26.5) | 9 (13.6) | 8 (12.1) | 2 (3) |  |  | 2 (3) | 19 (28.8) |
| Other AE |  |  |  |  |  |  |  | 2 (3) | 1 (1.5) | 1 (1.5) |  |  | 1 (1.5) | 4 (6.1) |
| METABOLISM AND NUTRITION DISORDERS |  |  |  |  |  |  |  |  |  |  |  |  |  |  |
| Anorexia | 4 (11.8) |  | 2 (5.9) |  |  | 2 (5.9) | 6 (17.6) | 11 (16.7) | 4 (6.1) | 6 (9.1) |  |  | 6 (9.1) | 21 (31.8) |
| Dehydration |  |  | 1 (2.9) |  |  | 1 (2.9) | 1 (2.9) |  |  |  |  |  |  |  |
| Hypercalcemia |  |  |  |  |  |  |  |  | 1 (1.5) |  |  |  |  | 1 (1.5) |
| Hyperglycemia | 1 (2.9) | 1 (2.9) |  |  |  |  | 2 (5.9) |  |  |  |  |  |  |  |
| Hypocalcemia |  |  | 1 (2.9) |  |  | 1 (2.9) | 1 (2.9) | 1 (1.5) |  |  |  |  |  | 1 (1.5) |
| Hypokalemia |  |  |  |  |  |  |  | 2 (3) |  |  |  |  |  | 2 (3) |
| Hyponatremia |  |  |  |  |  |  |  | 1 (1.5) |  |  |  |  |  | 1 (1.5) |
| Other AE |  |  |  |  |  |  |  | 1 (1.5) |  |  |  |  |  | 1 (1.5) |
| MUSCULOSKELETAL AND CONNECTIVE TISSUE DISORDERS |  |  |  |  |  |  |  |  |  |  |  |  |  |  |
| Arthralgia | 1 (2.9) |  |  |  |  |  | 1 (2.9) | 1 (1.5) |  |  |  |  |  | 1 (1.5) |
| Arthritis |  |  |  |  |  |  |  | 1 (1.5) |  |  |  |  |  | 1 (1.5) |
| Back Pain |  | 1 (2.9) | 1 (2.9) |  |  | 1 (2.9) | 2 (5.9) | 1 (1.5) |  | 1 (1.5) |  |  | 1 (1.5) | 2 (3) |
| Bone Pain | 1 (2.9) |  |  |  |  |  | 1 (2.9) | 2 (3) |  |  |  |  |  | 2 (3) |
| Chest Wall Pain |  | 1 (2.9) |  |  |  |  | 1 (2.9) |  |  |  |  |  |  |  |
| Flank Pain |  | 1 (2.9) |  |  |  |  | 1 (2.9) |  |  |  |  |  |  |  |
| Myalgia | 2 (5.9) | 1 (2.9) |  |  |  |  | 3 (8.8) | 1 (1.5) | 2 (3) |  |  |  |  | 3 (4.5) |
| Neck Pain | 1 (2.9) |  |  |  |  |  | 1 (2.9) |  |  |  |  |  |  |  |
| Pain In Extremity |  | 1 (2.9) |  |  |  |  | 1 (2.9) |  |  | 1 (1.5) |  |  | 1 (1.5) | 1 (1.5) |
| Other AE | 1 (2.9) |  |  |  |  |  | 1 (2.9) | 2 (3) |  | 1 (1.5) |  |  | 1 (1.5) | 3 (4.5) |
| NEOPLASMS BENIGN, MALIGNANT AND UNSPECIFIED (INCL CYSTS AND POLYPS) |  |  |  |  |  |  |  |  |  |  |  |  |  |  |
| Tumor Pain | 1 (2.9) | 1 (2.9) |  |  |  |  | 2 (5.9) |  |  | 1 (1.5) |  |  | 1 (1.5) | 1 (1.5) |
| NERVOUS SYSTEM DISORDERS |  |  |  |  |  |  |  |  |  |  |  |  |  |  |
| Depressed Level Of Consciousness |  |  | 1 (2.9) |  |  | 1 (2.9) | 1 (2.9) |  |  |  |  |  |  |  |
| Dizziness |  |  |  |  |  |  |  | 2 (3) | 1 (1.5) |  |  |  |  | 3 (4.5) |
| Dysgeusia | 2 (5.9) |  |  |  |  |  | 2 (5.9) |  |  |  |  |  |  |  |
| Headache | 1 (2.9) | 1 (2.9) |  |  |  |  | 2 (5.9) | 4 (6.1) | 2 (3) | 1 (1.5) |  |  | 1 (1.5) | 7 (10.6) |
| Intracranial Hemorrhage |  |  |  |  | 1 (2.9) | 1 (2.9) | 1 (2.9) |  |  |  |  | 1 (1.5) | 1 (1.5) | 1 (1.5) |
| Lethargy |  |  |  |  |  |  |  | 2 (3) | 2 (3) |  |  |  |  | 4 (6.1) |
| Paresthesia |  |  |  |  |  |  |  | 1 (1.5) |  |  |  |  |  | 1 (1.5) |
| Peripheral Motor Neuropathy |  | 1 (2.9) |  |  |  |  | 1 (2.9) | 1 (1.5) |  |  |  |  |  | 1 (1.5) |
| Radiculitis |  |  |  |  |  |  |  |  | 1 (1.5) |  |  |  |  | 1 (1.5) |
| Somnolence |  |  |  |  |  |  |  | 1 (1.5) |  |  |  |  |  | 1 (1.5) |
| Stroke |  |  |  |  | 1 (2.9) | 1 (2.9) | 1 (2.9) |  |  |  |  |  |  |  |
| Other AE |  | 1 (2.9) | 1 (2.9) |  |  | 1 (2.9) | 2 (5.9) | 2 (3) |  | 2 (3) |  |  | 2 (3) | 4 (6.1) |
| PSYCHIATRIC DISORDERS |  |  |  |  |  |  |  |  |  |  |  |  |  |  |
| Agitation |  |  |  |  |  |  |  | 1 (1.5) | 1 (1.5) |  |  |  |  | 2 (3) |
| Anxiety | 1 (2.9) |  |  |  |  |  | 1 (2.9) | 1 (1.5) |  |  |  |  |  | 1 (1.5) |
| Depression |  | 1 (2.9) |  |  |  |  | 1 (2.9) |  |  |  | 1 (1.5) |  | 1 (1.5) | 1 (1.5) |
| Insomnia | 1 (2.9) |  |  |  |  |  | 1 (2.9) |  |  |  |  |  |  |  |
| RENAL AND URINARY DISORDERS |  |  |  |  |  |  |  |  |  |  |  |  |  |  |
| Acute Kidney Injury |  |  |  |  |  |  |  |  |  |  | 1 (1.5) |  | 1 (1.5) | 1 (1.5) |
| Hematuria |  |  |  |  |  |  |  | 1 (1.5) |  |  |  |  |  | 1 (1.5) |
| Renal Colic |  |  |  |  |  |  |  |  | 1 (1.5) |  |  |  |  | 1 (1.5) |
| Urinary Retention |  | 1 (2.9) |  |  |  |  | 1 (2.9) |  |  |  |  |  |  |  |
| RESPIRATORY, THORACIC AND MEDIASTINAL DISORDERS |  |  |  |  |  |  |  |  |  |  |  |  |  |  |
| Allergic Rhinitis |  |  |  |  |  |  |  | 1 (1.5) |  |  |  |  |  | 1 (1.5) |
| Bronchopulmonary Hemorrhage |  |  |  |  |  |  |  |  | 1 (1.5) | 1 (1.5) |  |  | 1 (1.5) | 2 (3) |
| Cough |  |  |  |  |  |  |  | 1 (1.5) | 2 (3) |  |  |  |  | 3 (4.5) |
| Dyspnea | 3 (8.8) | 1 (2.9) | 2 (5.9) | 1 (2.9) |  | 3 (8.8) | 7 (20.6) | 5 (7.6) | 3 (4.5) | 1 (1.5) |  | 1 (1.5) | 2 (3) | 10 (15.2) |
| Pharyngeal Mucositis |  |  |  |  |  |  |  | 1 (1.5) |  |  |  |  |  | 1 (1.5) |
| Pleural Effusion |  | 1 (2.9) |  |  |  |  | 1 (2.9) | 1 (1.5) |  |  |  |  |  | 1 (1.5) |
| Pneumonitis |  |  |  |  |  |  |  |  | 1 (1.5) |  |  |  |  | 1 (1.5) |
| Other AE |  |  | 1 (2.9) |  |  | 1 (2.9) | 1 (2.9) |  | 1 (1.5) |  |  |  |  | 1 (1.5) |
| SKIN AND SUBCUTANEOUS TISSUE DISORDERS |  |  |  |  |  |  |  |  |  |  |  |  |  |  |
| Alopecia | 2 (5.9) |  |  |  |  |  | 2 (5.9) | 3 (4.5) |  |  |  |  |  | 3 (4.5) |
| Dry Skin | 5 (14.7) |  |  |  |  |  | 5 (14.7) | 1 (1.5) |  |  |  |  |  | 1 (1.5) |
| Pain Of Skin |  |  |  |  |  |  |  | 1 (1.5) |  |  |  |  |  | 1 (1.5) |
| Palmar-Plantar Erythrodysesthesia Syndrome | 1 (2.9) |  |  |  |  |  | 1 (2.9) | 1 (1.5) |  |  |  |  |  | 1 (1.5) |
| Pruritus | 1 (2.9) |  |  |  |  |  | 1 (2.9) | 2 (3) |  |  |  |  |  | 2 (3) |
| Rash Acneiform |  |  |  |  |  |  |  | 1 (1.5) |  |  |  |  |  | 1 (1.5) |
| Rash Maculo-Papular | 2 (5.9) |  |  |  |  |  | 2 (5.9) | 3 (4.5) |  |  |  |  |  | 3 (4.5) |
| Skin Induration | 1 (2.9) |  |  |  |  |  | 1 (2.9) |  |  |  |  |  |  |  |
| Urticaria |  |  |  |  |  |  |  | 1 (1.5) |  |  |  |  |  | 1 (1.5) |
| Other AE | 2 (5.9) |  |  |  |  |  | 2 (5.9) | 1 (1.5) | 2 (3) |  |  |  |  | 3 (4.5) |
| SURGICAL AND MEDICAL PROCEDURES |  |  |  |  |  |  |  |  |  |  |  |  |  |  |
| Other AE |  |  |  |  |  |  |  |  | 1 (1.5) |  |  |  |  | 1 (1.5) |
| VASCULAR DISORDERS |  |  |  |  |  |  |  |  |  |  |  |  |  |  |
| Flushing |  |  |  |  |  |  |  | 1 (1.5) |  |  |  |  |  | 1 (1.5) |
| Hypertension | 3 (8.8) | 3 (8.8) | 1 (2.9) |  |  | 1 (2.9) | 7 (20.6) | 5 (7.6) | 9 (13.6) | 4 (6.1) |  |  | 4 (6.1) | 18 (27.3) |
| Thromboembolic Event |  | 1 (2.9) |  | 2 (5.9) |  | 2 (5.9) | 3 (8.8) |  | 1 (1.5) |  |  | 1 (1.5) | 1 (1.5) | 2 (3) |
| Other AE |  |  |  |  |  |  |  | 1 (1.5) |  |  |  |  |  | 1 (1.5) |

Supplemental table 2: All adverse events, by treatment.

|  | **Placebo (Safety pop:N=34)** | | | | **Nintedanib (Safety pop:N=66)** | | | |
| --- | --- | --- | --- | --- | --- | --- | --- | --- |
| **System Organ Class + Preferred term** | **Grade  3  N (%)** | **Grade  4  N (%)** | **Grade  5  N (%)** | **Grade  ≥3  N (%)** | **Grade  3  N (%)** | **Grade  4  N (%)** | **Grade  5  N (%)** | **Grade  ≥3  N (%)** |
| PATIENTS' WORST GRADE | 8 (23.5) | 2 (5.9) | 2 (5.9) | 12 (35.3) | 30 (45.5) | 2 (3.0) | 3 (4.5) | 35 (53.0) |
| CARDIAC DISORDERS |  |  |  |  |  |  |  |  |
| Cardiac Arrest |  |  |  |  |  |  | 1 (1.5) | 1 (1.5) |
| Pericardial Effusion |  |  |  |  | 1 (1.5) |  |  | 1 (1.5) |
| GASTROINTESTINAL DISORDERS |  |  |  |  |  |  |  |  |
| Abdominal Pain |  |  |  |  | 2 (3) |  |  | 2 (3) |
| Diarrhea | 1 (2.9) |  |  | 1 (2.9) | 8 (12.1) |  |  | 8 (12.1) |
| Nausea |  |  |  |  | 4 (6.1) |  |  | 4 (6.1) |
| Vomiting | 1 (2.9) |  |  | 1 (2.9) | 1 (1.5) |  |  | 1 (1.5) |
| GENERAL DISORDERS AND ADMINISTRATION SITE CONDITIONS |  |  |  |  |  |  |  |  |
| Fatigue | 2 (5.9) |  |  | 2 (5.9) | 4 (6.1) |  |  | 4 (6.1) |
| Pain |  |  |  |  | 1 (1.5) |  |  | 1 (1.5) |
| HEPATOBILIARY DISORDERS |  |  |  |  |  |  |  |  |
| Cholecystitis |  |  |  |  | 1 (1.5) |  |  | 1 (1.5) |
| INFECTIONS AND INFESTATIONS |  |  |  |  |  |  |  |  |
| Arteritis Infective |  |  |  |  | 1 (1.5) |  |  | 1 (1.5) |
| Lung Infection |  |  |  |  | 1 (1.5) |  |  | 1 (1.5) |
| Sepsis |  | 1 (2.9) |  | 1 (2.9) |  |  |  |  |
| Other AE | 1 (2.9) |  |  | 1 (2.9) | 1 (1.5) |  |  | 1 (1.5) |
| INVESTIGATIONS |  |  |  |  |  |  |  |  |
| Alkaline Phosphatase Increased |  |  |  |  | 1 (1.5) |  |  | 1 (1.5) |
| Ggt Increased |  |  |  |  | 7 (10.6) |  |  | 7 (10.6) |
| Serum Amylase Increased |  |  |  |  | 1 (1.5) |  |  | 1 (1.5) |
| Weight Loss | 1 (2.9) |  |  | 1 (2.9) | 2 (3) |  |  | 2 (3) |
| Other AE |  |  |  |  | 1 (1.5) |  |  | 1 (1.5) |
| METABOLISM AND NUTRITION DISORDERS |  |  |  |  |  |  |  |  |
| Anorexia | 2 (5.9) |  |  | 2 (5.9) | 6 (9.1) |  |  | 6 (9.1) |
| Dehydration | 1 (2.9) |  |  | 1 (2.9) |  |  |  |  |
| Hypocalcemia | 1 (2.9) |  |  | 1 (2.9) |  |  |  |  |
| MUSCULOSKELETAL AND CONNECTIVE TISSUE DISORDERS |  |  |  |  |  |  |  |  |
| Back Pain | 1 (2.9) |  |  | 1 (2.9) | 1 (1.5) |  |  | 1 (1.5) |
| Pain In Extremity |  |  |  |  | 1 (1.5) |  |  | 1 (1.5) |
| Other AE |  |  |  |  | 1 (1.5) |  |  | 1 (1.5) |
| NEOPLASMS BENIGN, MALIGNANT AND UNSPECIFIED (INCL CYSTS AND POLYPS) |  |  |  |  |  |  |  |  |
| Tumor Pain |  |  |  |  | 1 (1.5) |  |  | 1 (1.5) |
| NERVOUS SYSTEM DISORDERS |  |  |  |  |  |  |  |  |
| Depressed Level Of Consciousness | 1 (2.9) |  |  | 1 (2.9) |  |  |  |  |
| Headache |  |  |  |  | 1 (1.5) |  |  | 1 (1.5) |
| Intracranial Hemorrhage |  |  | 1 (2.9) | 1 (2.9) |  |  | 1 (1.5) | 1 (1.5) |
| Stroke |  |  | 1 (2.9) | 1 (2.9) |  |  |  |  |
| Other AE | 1 (2.9) |  |  | 1 (2.9) | 2 (3) |  |  | 2 (3) |
| PSYCHIATRIC DISORDERS |  |  |  |  |  |  |  |  |
| Depression |  |  |  |  |  | 1 (1.5) |  | 1 (1.5) |
| RENAL AND URINARY DISORDERS |  |  |  |  |  |  |  |  |
| Acute Kidney Injury |  |  |  |  |  | 1 (1.5) |  | 1 (1.5) |
| RESPIRATORY, THORACIC AND MEDIASTINAL DISORDERS |  |  |  |  |  |  |  |  |
| Bronchopulmonary Hemorrhage |  |  |  |  | 1 (1.5) |  |  | 1 (1.5) |
| Dyspnea | 2 (5.9) | 1 (2.9) |  | 3 (8.8) | 1 (1.5) |  | 1 (1.5) | 2 (3) |
| Other AE | 1 (2.9) |  |  | 1 (2.9) |  |  |  |  |
| VASCULAR DISORDERS |  |  |  |  |  |  |  |  |
| Hypertension | 1 (2.9) |  |  | 1 (2.9) | 4 (6.1) |  |  | 4 (6.1) |
| Thromboembolic Event |  | 2 (5.9) |  | 2 (5.9) |  |  | 1 (1.5) | 1 (1.5) |
